# Potential Toxicity of Nine Rare Earth Elements (REEs) on Marine Copepod *Tigriopus fulvus*

**DOI:** 10.3390/jox14040102

**Published:** 2024-12-04

**Authors:** Francesca Biandolino, Ermelinda Prato, Asia Grattagliano, Giovanni Libralato, Marco Trifuoggi, Isabella Parlapiano

**Affiliations:** 1 National Research Council, Water Research Institute (IRSA-CNR), Via Roma, 3, 74123 Taranto, Italy; francesca.biandolino@irsa.cnr.it (F.B.); isabella.parlapiano@irsa.cnr.it (I.P.); 2 Department of Chemical Sciences and Technologies, University of Rome “Tor Vergata”, Via della Ricerca Scientifica, 1, 00133 Roma, Italy; asia.grattagliano@uniroma2.it; 3 Department of Biology, University of Naples Federico II, Via Cinthia 21, 80126 Naples, Italy; giovanni.libralato@unina.it; 4 Department of Chemical Sciences, University of Naples Federico II, Via Cinthia 21, 80126 Naples, Italy; marco.trifuoggi@unina.it

**Keywords:** rare earth elements (REEs), potential impact, marine copepod, acute mortality tests

## Abstract

The present study focused, for the first time, on the adverse effects of nine REEs on the marine copepod *Tigriopus fulvus.* For this purpose, copepod mortality, immobilization, and naupliar development were assessed. Overall, the results demonstrated that all REEs tested exerted significant adverse effects on *T. fulvus*, with LC50 values ranging from 0.56 to 1.99 mg/L. Concentration-dependent increases in mortality and immobilization for all tested REEs were observed. Following exposure of nauplii to REEs, a significant slowing of nauplii development was shown with all REEs tested. The results obtained clearly highlight the potential toxicity of REEs, and, in particular, of Lanthanum, which could have consequences on the survival and development of *T. fulvus*, affecting the copepod population.

## 1. Introduction

Technological progress in recent decades have led to an increase in demand for rare earth elements (REEs). This group of seventeen metals, including Scandium, Yttrium, and lanthanides, has many extraordinary physico-chemical, catalytic, electrical, magnetic, and optical properties, making them essential in fields ranging from smart and green technologies to raw materials for industrial applications [1,2]. In modern technologies such as mobile phones, televisions, LED light bulbs, and wind turbines, hybrid automobiles, medical devices, hard drives, and defence technologies, they play a key role [3].

Despite their name, these elements are not ‘rare’ [4], but most of the reserves of these elements are not economically exploitable and their separation is difficult due to their similar chemical properties [5]. China currently holds a dominant position in the global production of REEs (70%), as it has the largest reserves of REEs, followed by the US (14.3%) and Australia (6%) [6].

REEs occur together in natural systems, as minor or major constituents, rather than as individual native metals, and sites in mining-impacted areas have been found to contain REEs at concentrations up to 100 times normal background levels. Therefore, the risks associated with the extraction and production of REEs concern not only the products but also the REE-containing wastes generated during production. These wastes increase the concentration of REEs in the surface environment and have the potential to alter the natural physico-chemical conditions of the soil and water (e.g., pH and redox states). Acidity can lead to the solubilization of otherwise stable REE fractions, thereby increasing their bioavailability and migration into the environment. As a result, high concentrations of REEs have been detected in soils, vegetables and other crops, and even in human bodies, near extraction and processing sites [7,8,9,10,11].

The increased use of REEs combined with their inadequate removal by wastewater treatment plants increases the risk of these substances entering aquatic environments [12,13], with consequent potential hazard to aquatic organisms [14,15]. Gadolinium (Gd) concentrations of up to 1100 mg/L have been found in river discharges from wastewater treatment plants [16], while concentrations of up to 200 ng/L have been found in unpolluted rivers [17,18]. Lanthanum (La) has been reported at concentrations from 0.029 to 12.85 μg/L in surface waters and up to 40.37 μg/L in alluvial aquifers affected by acid mine drainage [19,20,21]. The concentrations of La and cerium (Ce) in wastewater range from 7.7 to 80.4 μg/L and from 19.4 to 161 μg/L, respectively [22]. In river water, La and Ce concentrations are lower than in wastewater, ranging from 19.7 to 74 ng/L and from 9.67 to 212 ng/L, respectively [22], but on a local basis, they can range from 80 to 200 μg/L, respectively [23].

The understanding of REEs’ mechanisms of action in biological systems is not yet fully clear [24]. Recent studies have focused on the adverse effects of REEs on aquatic organisms such as fish and molluscs, demonstrating that REEs can be absorbed and accumulated in organic tissues [25,26]. This may lead to an increase in reactive oxygen species (ROS), inhibition of the antioxidant system and damage to the DNA of exposed aquatic organisms [27,28]. Most extant research has investigated the toxicity of few REEs (e.g., Gd, La, Ce) on freshwater organisms [24,29,30,31]. So far, data on the REEs’ toxicity effects on marine organisms are still limited, mostly derived from acute and chronic toxicity testing on bivalve molluscs, sea urchins, and unicellular algae [32,33,34,35,36,37]. Given the increasing use of REEs and their presence in the environment, especially in rivers and coastal areas, there is a need to monitor their potential effects on living biota, including bioassays on model organisms.

The harpacticoid copepod *Tigriopus fulvus* is a commonly used model organism for acute toxicity testing due to its ease of breeding in the laboratory and its year-round availability. It has a high sensitivity to a wide range of toxicants and the results of the related tests are reproducible [38].

The aim of this work was to give insight into the potential toxicity of nine REEs: scandium (Sc), yttrium (Y), lanthanum, (La), cerium (Ce), neodymium (Nd), gadolinium (Gd), dysprosium (Dy), holmium (Ho), and erbium (Er) on the marine copepod *T. fulvus*. Comparisons between the toxicity of the nine elements and their effects on mortality, immobility, and naupliar development were assessed.

## 2. Materials and Methods

### 2.1. Preparation of Test Medium, Rare Earth Elements (REE)

Test solutions were freshly prepared before each experiment by diluting commercial analytical grade standard solutions (1 g L^−1^) of Nd, Ho, Er, Dy, Sc, Y, Ce, La, and Gd (purity > 99%, Agilent, Italy) to the final exposure concentrations by using filtered natural seawater (FNSW, 0.22 μm, 38 PSU). Since the initial acid pH of the standard solutions was outside the tolerance range of the test organism, all pH values of the test solutions were measured with a pH metre (Mettler Toledo Five Easy, Milan, Italy) and adjusted to 8.0 ± 0.5 using NaOH prior to the assays.

For each REE, seven different test solutions in a geometric nominal concentration series with a factor of two (0.10, 0.20, 0.40, 0.80, 1.60, 3.20, and 6.40 mg REE/L) were tested.

Actual concentrations were determined by Inductively Coupled plasma mass spectrometry (ICP-MS, Aurora Bruker M90, Bremen, Germany) according to an established protocol [35]. Triplicate analyses were carried out.

### 2.2. Experimental Design and Conditions

Test organisms were obtained from a massive culture maintained in filtered natural seawater (FNSW, 0.45 μm) at a salinity of 38 PSU in a temperature-controlled room at 20 ± 2 °C, with cool light (500–1200 lux, Ostram Fluora, L58W/77; Milano, Italy) and a photoperiod cycle of 16 h light and 8 h dark. Tetramarin^®^ (fish food) was mixed with the microalgae *Tetraselmis suecica* and *Isochrysis galbana* to provide a weekly ad libitum diet for the copepods.

### 2.3. Test Procedures

All tests were carried out on newborns (nauplii ≤ 24 h-old) of *T. fulvus* following protocols reported by Faraponova et al. [38] and UNICHIM [39], in 12-well culture plates; each well contained 10 nauplii in 3 mL of test solution and a negative control (with only FNSW). Three replicates for each concentration and the negative control were performed. Plates were incubated for 96 h under the same conditions described above for culture maintenance without feeding.

The organism’s sensitivity and test validity were confirmed by conducting acute tests with the reference compound copper sulphate (CuSO_4_ × 5 H_2_O) (positive control).

During the entire duration of the test, the pH (8.0 ± 1.0), salinity (38.0 ± 1.0), and oxygen levels (>80% of saturation) of the solutions were checked.

The measured endpoints were mortality, immobility, and number of naupliar moults.

At the end of the tests, the number of dead and immobile nauplii was counted under a stereomicroscope. When nauplii failed to move any appendages after gentle stimulation for at least 20 s of observation, they were considered dead. The number of immobile nauplii was determined by summing the dead nauplii to those still living, not shifting their barycenter, but moving their appendages.

In addition, the effect of REE on the moulting process of *T. fulvus* was evaluated. The number of exuviae (moults) released during the 96 h test was counted. Crystal violet solution (Sigma-Aldrich, Cas 548-62-9) for microscopy was diluted 1:10 with distilled water. For each chamber test, a drop was added with a Pasteur pipette, to make the exuviae more visible under the stereomicroscope. All tests were replicated three times.

### 2.4. Data Analyses

All results are expressed as the mean ± standard deviation (SD) (*n* = 3). The median lethal concentration (LC50) and LC20 and, for immobility, the median effect concentration EC50 and EC20 with relative 95% confidence intervals (C.I.), were calculated using the Litchfield–Wilcoxon method. A smooth function was used to fit concentration-response curves with the GraphPad Prism 8.4.2 programme. The data were examined for normality and homogeneity of variances by Kolmogorov–Smirnov and Bartlett’s tests. When both assumptions were satisfied, differences between treatments were assessed via one-way analysis of variance (ANOVA). The post hoc Tukey’s test was used to account for differences within groups, setting the statistical significance at *p* < 0.05. All statistical analyses were conducted with the package software Past3 (version 1.0; Hammer, Norway).

## 3. Results and Discussion

### 3.1. Chemical Data

Table 1 shows the nominal and measured concentrations of the test solutions determined with the ICP-MS. The ratios of measured to nominal concentrations fell within a very narrow range from 0.64 to 1.22, highlighting that the nominal concentration values were always quite close to the measured ones. Therefore, nominal concentration values were considered reliable.

### 3.2. Acute Toxicity Test

The mean percentage of survival in the negative controls was >90% in each experiment, while the LC50 of the positive control was equal to 0.13 (0.07–0.18) mg/L of Cu^2+^, within the range of the laboratory control chart for this species, and in compliance with the acceptance criteria [40].

In general, the results from this study demonstrated that all tested REEs exerted significant adverse effects on *T. fulvus*. La and Gd exhibited a greater toxicity to *T. fulvus*, with LC50s of 0.56 and 0.92 mg/L, respectively (*p* < 0.05, Table 2), followed by Nd and Ho, which showed similar toxicity. Er was the least toxic at 1.99 mg/L (*p* < 0.05, Table 2). According to LC50s obtained, REEs were ranked in the decreasing order of toxicity as follows: La > Gd > Nd > Ho > Y > Sc > Ce > Dy > Er; therefore, REEs did not show a predictable pattern consistent with their atomic number. Most data on REEs’ toxicity to aquatic organisms are derived by acute toxicity tests, although their relative toxicity is often contradictory; therefore, it is unclear whether there is a common pattern of biological effect among all REEs. Some authors reported similar toxicity among REEs [30,41,42,43], likely due to similar physico-chemical properties influencing chemical behaviour and toxicity mechanisms. Conversely, other studies have shown that each REE has different effects, with different ecotoxicological risks among them. This makes it hard to identify their universal mode of toxicity [24,29,44,45].

Siciliano et al. [46] reported, for *Phaeodactylum tricornutum* exposed to REE values of LC50 from 0.98 to 13.21 mg/L, that the elements’s toxicity was ranked as Gd > Ce > Er > La > Eu > Nd > Dy > Sm. Pagano et al. [34], in a study on the effects of several REEs on *Paracentrotus lividus* embryos, reported the following ranking of toxicity: Gd > Y > La > Nd > Eu > Ce > Sm. On the other hand, no significant differences in the sensitivity of the diatom *Skeletonema costatum* between REEs were observed by Tai et al. [37].

Markich et al. [47] determined the chronic REEs toxicity based on 30 coastal marine organisms, reporting the following ranking: Y~Gd > Lu~Nd~Dy~Ce > La~Pr. As can be seen from the literature, Gd always seems to be one of the most toxic. This is a worrying fact, considering that Gd is the most used worldwide due to the widespread use of Gd-based contrast agents (GBCAs) in medical imaging, and their poor removal efficiency (<10%) by wastewater treatment plants. Indeed, Gd is the most prevalent REE contaminant in coastal marine waters.

The major cause of REE toxicity is the disturbance of Ca metabolism and homeostasis, damaging cells and reducing development, growth, reproduction and survival. This explains why the organisms, which require Ca to build shells, skeletons, or tests, are particularly sensitive to REEs, as reported by Markich et al. [47] for the sea urchin *Heliocidaris tuberculata*.

As in the current study, Borgmann et al. [48] reported for *Hyalella azteca* after one-week exposures to REEs LC50 values between 0.51 and 1.67 mg/L, although in the following decreasing toxicity order: Nd > Y > Gd > Ce > Ho > Dy > Er > La.

Blinova et al. [30] for *Thamnocephalus platyurus* reported LC50 values of 34.6, 33.0, and 31.80 mg/L for La, Ce, and Nd, respectively, while Manusadžianas et al. [49], in a study on 11 lanthanides and Y, found 24 h LC50 values ranging from 3.22 mg/L for Y to 45.2 mg/L for La. Blaise et al. [29], in a study on *Hydra attenuata* exposed to 11 REEs, reported LC50 values in the range 0.22–0.69 mg/L, which is lower compared with results of this study. However, if immobilization, which correlates closely with death, is considered as the endpoint, the values we identified are comparable with those observed by Blaise et al. [29]. The EC50 values for *T. fulvus* were included between 0.15 and 0.71 mg/L (Table 3), very similar to those reported by Blaise et al. [29]. Moreira et al. [50] reported the high sensitivity of oyster embryo-larval development, with EC50s of 0.15 mg/L after 24 h and 0.22 mg/L after 48 h exposure to Y.

Not surprisingly, sensitivity to REEs differs greatly among organisms and among the endpoints used, making comparisons difficult. In a toxicity study of seven REEs (yttrium, lanthanum, cerium, neodymium, samarium, europium, and gadolinium) on three echinoderm species, Trifuoggi et al. [51] found that *Sphaerechinus granularis* had a significantly higher sensitivity to Y and La (EC50 of 0.042 mg/L and 0.008 mg/L, respectively) than *Arbacia lixula* and *Paracentrotus lividus*. Moreover, *P. lividus* embryos exposed to Gd showed higher sensitivity (EC50 = 0.031 mg/L) than *A. lixula* and *S. granularis*. Also, Martino et al. [52] observed different sensitivities to Gd in a study on the sea urchin embryonic development of the European (*Paracentrotus lividus* and *Arbacia lixula*) and Australian species (*Heliocidaris tuberculate* and *Centrostephanus rodgersii*), suggesting that it may be toxic to marine organisms even within the same taxonomic group.

**Figure 1:**
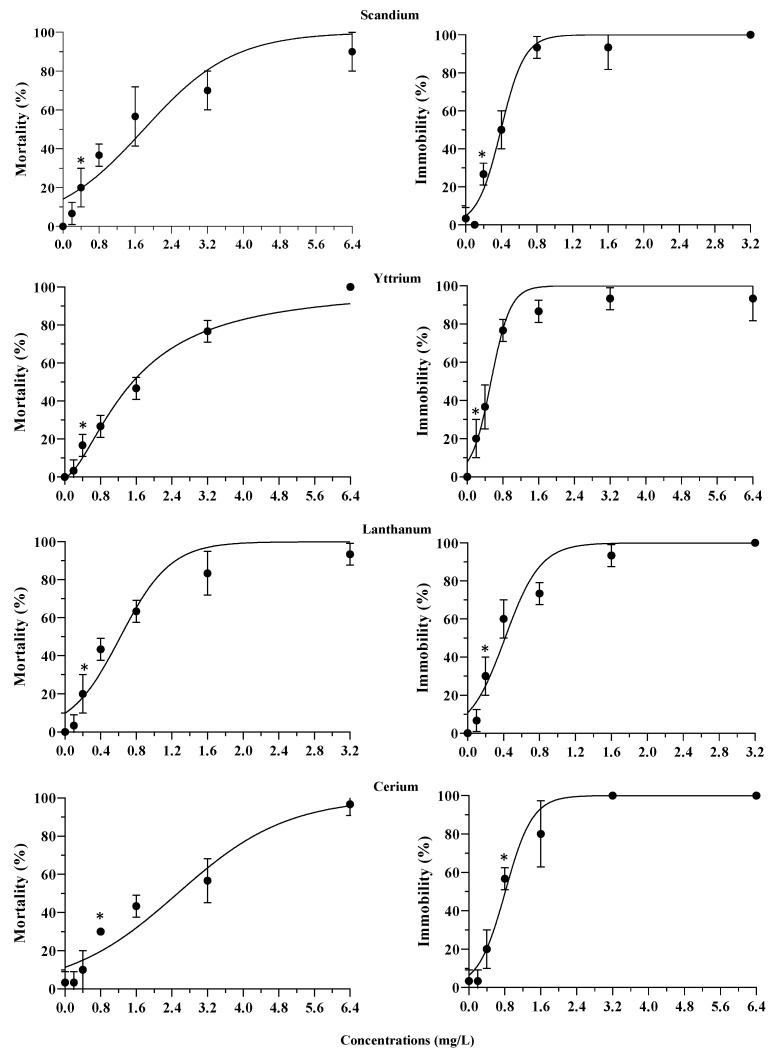
Concentration-response curves based on the mean (±s.d.; *n* = 30) mortality and immobilization rates of *T. fulvus* exposed to scandium, yttrium, lanthanum, and cerium. Asterisks indicate the first concentration causing significant differences to the control (Tukey’s test, *p* < 0.05).

**Figure 2:**
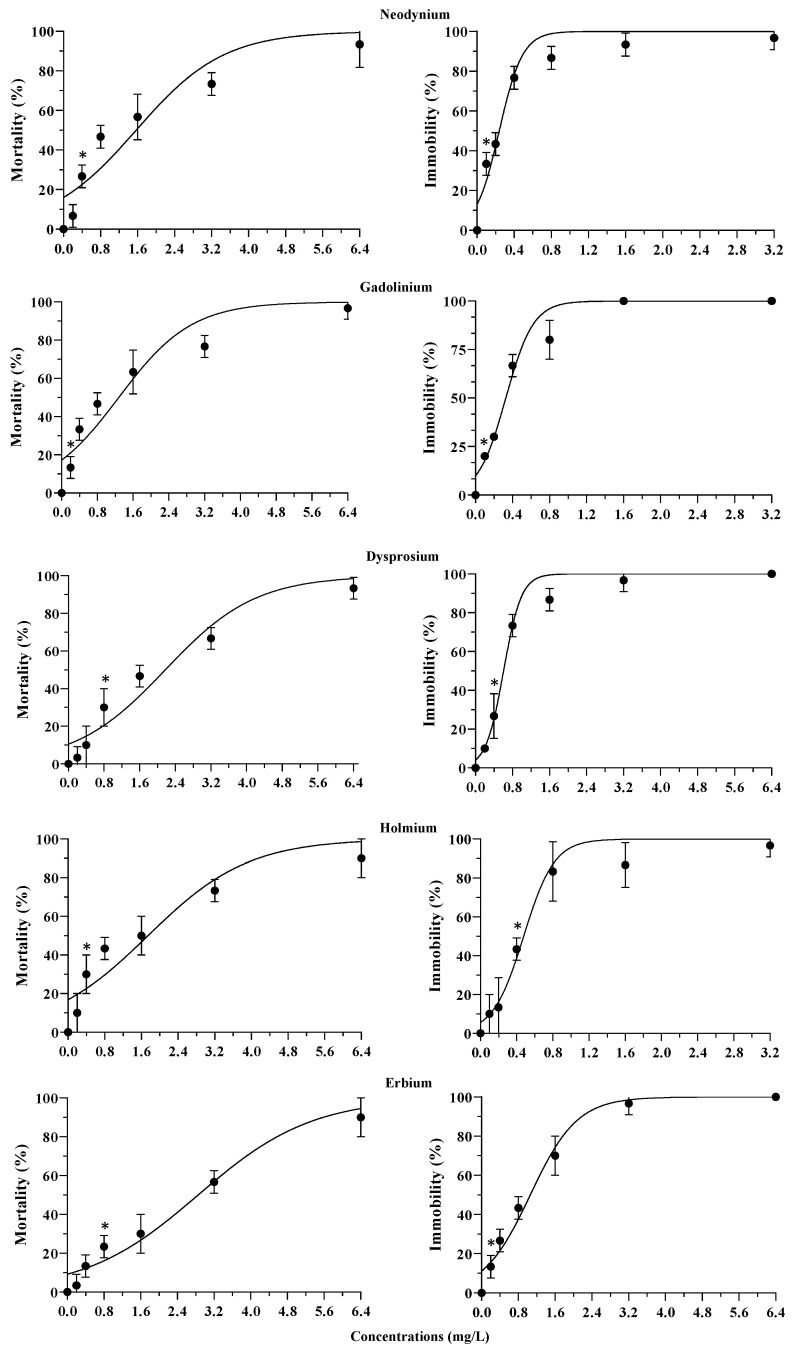
Concentration-response curves based on the mean (±s.d.; *n* = 30) mortality and immobilization rates of *T. fulvus* exposed to neodymium, gadolinium, dysprosium, holmium, and erbium. Asterisks indicate the lowest concentrations which show significant differences with the controls (*p* < 0.05).

Exposure to REEs resulted in concentration-dependent increases in mortality and immobilization for all REEs tested (Figure 1 and Figure 2). Most of the REEs showed a significant acute toxic effect compared to the control, even at the lowest concentrations, confirming a toxicity that should not be underestimated. Concerning *T. fulvus* exposure to La and Gd, the effects (mortality) ranged between 20% (0.2 mg/L) and 93% (3.2 mg/L) for La, and between 13% (0.2 mg/L) and 97% (6.4 mg/L) for Gd. For Er, the mortality varied between 3% (0.2 mg/L) and 90% (6.4 mg/L). As regards immobilization, exposure to Gd showed the effect varying between 20% (0.1 mg/L) and 100% (1.6 mg/L) (Figure 1 and Figure 2).

**Figure 3:**
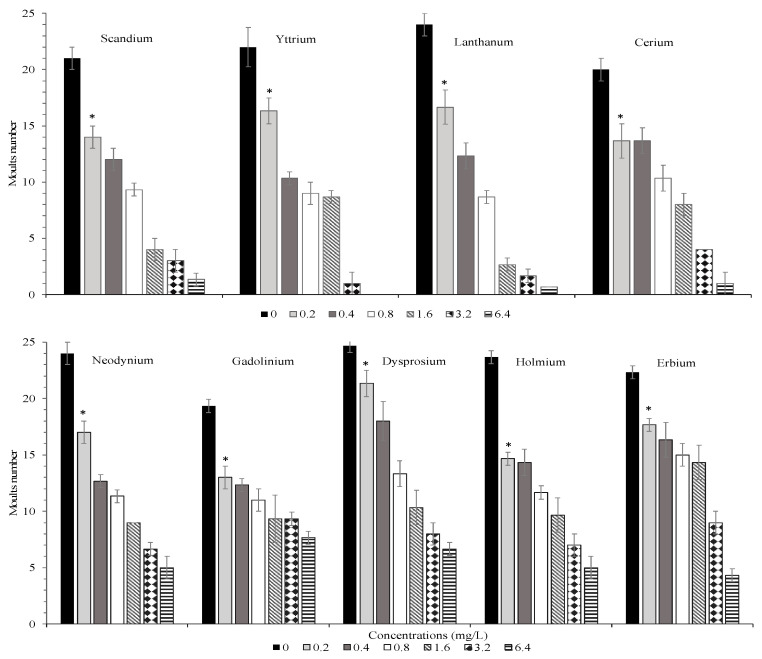
Moult numbers (mean ± s.d.) of *T. fulvus* exposed to different concentrations of REEs. Asterisks indicate the lowest concentrations which show significant differences with the controls (*p* < 0.05).

Furthermore, all REEs negatively affected the larval development of *T. fulvus.* Exposure to all REEs resulted in a concentration-dependent decrease in the number of naupliar moults, which was significant compared to the control even at the lowest concentration tested (0.2 mg/L). (Figure 3, *p* < 0.05). At the end of the test, a total of 20–25 naupliar moults were found in the negative control. After exposure of nauplii to REEs, a significant slowdown of naupliar development was exhibited by all tested REEs. However, at the highest concentration of 6.4 mg/L, a 100% reduction in naupliar moults was observed only in copepods exposed to Y, displaying the most severe sublethal effect compared to the other REEs. This sub-lethal effect may be a potential harmful signal, affecting individual fitness and overall reproductive output, offering insight into the future health of a population.

## 4. Conclusions

The continuous release of REEs into the marine environment poses a threat to marine species.

This study analyzed the acute and sub-lethal effects of nine REEs on the copepod *T. fulvus*. It is necessary to emphasize the importance of sub-lethal effects in ecotoxicological risk assessment, as reduced embryonic development and survival may pose a significant threat to adult recruitment and population health.

The results show more severe effects for La, Gd, and Nd on the copepod for all endpoints analyzed, while Er was the least toxic. Overall, a significant difference emerged between control copepods and those exposed to REEs even at the lowest concentrations. Although for some REEs, the LC50 and EC50 values obtained are outside the range of concentrations found in the natural environment, it must be considered that, given the role of REEs in our daily lives, their increasing demand, and consequent release into the environment, the results of this study can help to improve our understanding of the environmental impact of REEs and contribute to the definition of future guideline values and ecological risk calculations. In addition, further studies on chronic exposure to REE are needed to assess their effects on all life stages, as this could lead to significant changes.

## Figures and Tables

**Table 1:** Limit of Detection (LOD) and Limit of Quantitation (LOQ) values are expressed in µg/L. Nominal and measured REE concentrations mg/L (mean ± s.d.; *n* = 3).

		Nominal Concentrations (mg/L)						
		0.1	0.2	0.4	0.8	1.6	3.2	6.4
	LOD–LOQ (µg/L)	Measured concentrations (mg/L)						
**Sc**	0.066–0.220	0.08 ± 0.00	0.19 ± 0.01	0.36 ± 0.02	0.70 ± 0.02	1.20 ± 0.04	2.86 ± 0.06	5.57 ± 0.20
**Y**	0.003–0.010	0.09 ± 0.00	0.22 ± 0.02	0.39 ± 0.05	0.71 ± 0.02	1.45 ± 0.05	2.86 ± 0.03	5.89 ± 0.15
**La**	0.003–0.009	0.11 ± 0.01	0.18 ± 0.001	0.29 ± 0.002	0.94 ± 0.01	1.02 ± 0.00	3.43 ± 0.01	5.84 ± 0.09
**Ce**	0.003–0.011	0.09 ± 0.00	0.19 ± 0.01	0.37 ± 0.01	0.77 ± 0.03	1.48 ± 0.02	2.97 ± 0.05	5.60 ± 0.10
**Nd**	0.004–0.013	0.11 ± 0.00	0.17 ± 0.00	0.26 ± 0.01	0.61 ± 0.02	1.34 ± 0.01	2.70 ± 0.04	5.04 ± 0.10
**Gd**	0.003–0.009	0.08 ± 0.01	0.21 ± 0.03	0.38 ± 0.02	0.75 ± 0.04	1.60 ± 0.05	3.01 ± 0.06	5.79 ± 0.20
**Dy**	0.002–0.005	0.1 ± 0.01	0.21 ± 0.01	0.47 ± 0.03	0.93 ± 0.05	1.77 ± 0.10	3.72 ± 0.20	5.99 ± 0.20
**Ho**	0.002–0.006	0.12 ± 0.01	0.23 ± 0.01	0.42 ± 0.02	1.14 ± 0.05	1.12 ± 0.00	2.86 ± 0.08	6.40 ± 0.38
**Er**	0.001–0.005	0.12 ± 0.01	0.18 ± 0.01	0.33 ± 0.02	0.92 ± 0.01	1.10 ± 0.05	2.65 ± 0.01	5.73 ± 0.05

**Table 2:** LC20 and LC50 (mg/L) with 95% C.I., of *T. fulvus* exposed to REEs. Different letters within each column indicate significant differences (*p* < 0.05).

REEs	LC50	C.I. (95%)	LC20	C.I. (95%)
Scandium	1.33 ^bc^	1.85–0.95	0.44 ^c^	0.50–0.39
Yttrium	1.17 ^ab^	1.46–0.93	0.56 ^d^	0.61–0.50
Lanthanum	0.56 ^a^	0.74–0.43	0.23 ^a^	0.25–0.21
Cerium	1.53 ^bc^	2.08–1.13	0.63 ^de^	0.71–0.56
Neodynium	1.12 ^ab^	1.54–0.82	0.39 ^bc^	0.44–0.35
Gadolinum	0.92 ^ab^	1.26–0.67	0.32 ^ab^	0.36–0.28
Dysprosium	1.56 ^bc^	2.15–1.13	0.62 ^de^	0.69–0.56
Holmium	1.15 ^ab^	1.67–0.79	0.33 ^b^	0.38–0.29
Erbium	1.99 ^c^	2.86–1.38	0.70 ^e^	0.80–0.61

**Table 3:** EC20 and EC50 (mg/L) with 95% C.I., of immobilization of *T. fulvus* exposed to REEs. Different letters within each column indicate significant differences (*p* < 0.05).

REEs	EC50	C.I. (95%)	EC20	C.I. (95%)
Scandium	0.57 ^cde^	0.69–0.47	0.36 ^de^	0.38–0.35
Yttrium	0.49 ^cd^	0.67–0.36	0.20 ^b^	0.23–0.18
Lanthanum	0.37 ^bc^	0.51–0.27	0.17 ^b^	0.19–0.16
Cerium	0.74 ^e^	0.89–0.61	0.40 ^e^	0.42–0.37
Neodynium	0.19 ^ab^	0.28–0.12	0.06 ^a^	0.06–0.05
Gadolinum	0.15 ^a^	0.20–0.12	0.07 ^a^	0.08–0.06
Dysprosium	0.60 ^de^	0.75–0.48	0.28 ^c^	0.31–0.26
Holmium	0.44 ^cd^	0.58–0.34	0.19 ^b^	0.21–0.17
Erbium	0.71 ^e^	0.90–0.56	0.33 ^d^	0.36–0.30

## Data Availability

The original contributions presented in the study are included in the article, further inquiries can be directed to the corresponding authors.
